# Combinatorial Communication in Bacteria: Implications for the Origins of Linguistic Generativity

**DOI:** 10.1371/journal.pone.0095929

**Published:** 2014-04-23

**Authors:** Thomas C. Scott-Phillips, James Gurney, Alasdair Ivens, Stephen P. Diggle, Roman Popat

**Affiliations:** 1 Department of Anthropology, Durham University, Durham, United Kingdom; 2 School of Life Sciences, University of Nottingham, Nottingham, United Kingdom; 3 Centre for Immunity, Infection and Evolution, University of Edinburgh, Edinburgh, United Kingdom; University of York, United Kingdom

## Abstract

Combinatorial communication, in which two signals are used together to achieve an effect that is different to the sum of the effects of the component parts, is apparently rare in nature: it is ubiquitous in human language, appears to exist in a simple form in some non-human primates, but has not been demonstrated in other species. This observed distribution has led to the pair of related suggestions, that (i) these differences in the complexity of observed communication systems reflect cognitive differences between species; and (ii) that the combinations we see in non-human primates may be evolutionary pre-cursors of human language. Here we replicate the landmark experiments on combinatorial communication in non-human primates, but in an entirely different species, unrelated to humans, and with no higher cognition: the bacterium Pseudomonas aeruginosa. Using the same general methods as the primate studies, we find the same general pattern of results: the effect of the combined signal differs from the composite effect of the two individual signals. This suggests that advanced cognitive abilities and large brains do not necessarily explain why some species have combinatorial communication systems and others do not. We thus argue that it is premature to conclude that the systems observed in non-human primates are evolutionarily related to language. Our results illustrate the value of an extremely broad approach to comparative research.

## Introduction

**Figure 1:**
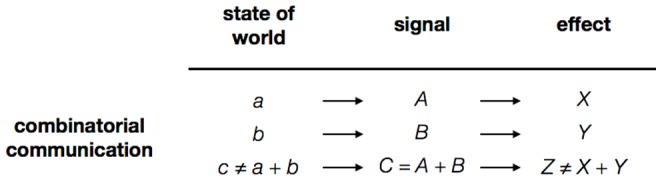
Combinatorial communication. In a combinatorial communication system, two (or more) holistic signals (A and B in this figure) are combined to form a third, composite signal (A+B), which has a different effect (Z) to the sum of the two individual signals (X+Y). Applied to the putty-nosed monkey system, the symbols in this figure would be: a =  presence of eagles; b =  presence of leopards; c =  absence of food; A =  ‘pyow’; B =  ‘hack’ call; C = A+B =  ‘pyow-hack’; X =  climb down; Y =  climb up; Z≠X+Y =  need for group movement (e.g. to forage). Several systems in nature (e.g. the waggle dance of honeybees) have signals composed of two distinct parts, but this composite signal is not different to the sum of the component parts (in the terms of this figure, there is a state of the world c = a+b, with a signal C = A+B and a response Z = X+Y).

Many species have communication systems in which two (or more) signals are produced alongside one another. This is the case with honeybee dance, for example, where one part of the dance describes direction, and another distance [1]. However, there are far fewer natural communication systems that are known to be properly combinatorial, in the sense that two signals are produced together to achieve an effect that is different to the sum of the effects of the component parts (figure 1) [2]–[4]. This combinatorial quality is clearly present in human language, in which composite signals are not just present, but ubiquitous. Indeed, they are what gives language its expressive power [4], [5], and are the reason why the origins of language is considered one of just eight major transitions in the evolution of life [6]. Outside of language, the most well-known evidence of combinatorial communication is the alarm call system of putty-nosed monkeys, which is reported to include a distinct call for each of two predators, and also a call sequence that appears to involve the two distinct calls being produced together, but with an effect that is not simply the composite effect of the two component parts, but something different, namely group travel in non alarm situations [7], [8]. The predator calls are often simply glossed as ‘pyow’ for leopards and ‘hack’ for eagles, although the data are in fact not as clean as this, and instead operate probabilistically. Outside of primates, combinatorial communication is currently unknown in the natural world [3], [4].

This observed distribution of combinatorial communication - ubiquitous in language, simple in non-human primates, and otherwise unknown in the natural world - invites the suggestion that these differences in the complexity of observed communication systems reflect, in some more-or-less uniform way, cognitive differences between species. This in turn leads to the assumption that the combinations we see in non-human primates are evolutionary pre-cursors of human language: the very smallest beginnings of morphosyntax [4], [9], [10]. However such conclusions are premature, since we have not thoroughly explored whether similar systems exist in other species. Evidence that the communication system of a species with limited cognitive abilities has the general properties described in figure 1 would undermine these arguments.

Here we implement the same general methods as those used in primate communication research, but in a species that is unrelated to primates, and which has no higher cognitive abilities: the bacterium Pseudomonas aeruginosa. Like many species of bacteria, P. aeruginosa communicates by quorum sensing (QS). Individual cells produce small diffusible signal molecules, and monitor the concentration of these molecules in the local environment. Specifically, the QS molecule binds to its cognate receptor forming a transcriptional factor which regulates the expression of QS-dependent genes [11]. In this way, QS allows individual cells to communicate their presence to other cells, and hence allows groups of cells to act in a population density dependent manner [12]–[14]. QS has been extensively studied in P. aeruginosa, where a number of distinct signalling molecules have been identified, and which have been shown to regulate a number of phenotypes including toxin production important for virulence [12]. QS satisfies standard biological definitions of communication [15], [16]: the purported signals cause specific effects in other individuals, and both production and reception is adapted to this purpose [14], [17]. In particular, the two signal molecules we use in this study (see below) have both been shown to satisfy these criteria [14].

## Background and General Methods

We focus on the two chemically related N-acyl homoserine lactones, N-(3-oxododecanoyl)-L-homoserine lactone and N-(butanoyl)-L-homoserine lactone (hereafter 3-oxo-C12-HSL and C4-HSL respectively). These signalling molecules are synthesised via distinct synthase proteins (LasI and RhlI) and interact with different receptor proteins (LasR and RhlR) forming two distinct receptor signal complexes [18], [19]. Although they are chemically distinct and act via different receptors, they are produced together in vitro and found together in vivo [20], [21]. Their regulatory effect in P. aeruginosa has previously been studied via transcriptomic microarray, targeted and transposon mutagenesis studies, and in vitro overexpression [22]–[26]. These studies show that each signal independently regulates a set of genes (some of which overlap), and that C4-HSL modulates the effect of 3-oxo-C12-HSL. It was previously thought that the two QS systems (lasRI and rhlRI) were organised in a hierarchical manner and therefore that the rhlRI system could have no activity in the absence of the lasRI system [27], [28], but it has since been shown that the rhlRI system can have activity independent of the lasRI system and that the mechanistic architecture of the two systems is not strictly hierarchical [29], [30].

Here we extend this previous work with a fully factorial experimental design, in which we expose the bacteria to both signals individually and to their combination. This allows us to detect any synergy in response to the two-signal combination. As such, our methods and design are strictly analogous to the playback experiments used to study non-human primate communication: the population is artificially exposed to previously identified, naturally occurring signals and their combination (and to appropriate controls), and the responses are observed. Specifically, we exposed P. aeruginosa signal-negative strain, unable to produce its own signals, to four different signalling conditions: two with separate, individual signal molecules; one with the combination of the same two molecules; and a baseline condition of no signal molecule (see Methods & Materials for details). This focus on combinatorial communication is appropriate for two main reasons. First, combinatorial communication is clearly a pre-requisite for any of the more complex aspects of natural language syntax, in particular semantically compositional syntax, which may be unique to human language [see 4 for discussion]. Second, combinatorial communication has, as discussed, been reported in the communication systems of other non-human species, and as such our results allow for direct comparisons with existing data.

## Results

Our null hypothesis about what the composite effect of the two individual signals would be if there was no effect of combining the two signals is that the effects of the combination should be equal to the sum of the individual effects. To measure this we subtracted the sum of effects that each individual signal had on its own from the effects that the two signals had in conjunction with one another. If the effect of the combined signal differs from the composite effect of the two individual signals, then the net result of this subtraction should differ from the effect of the signal combination.

**Figure 2:**
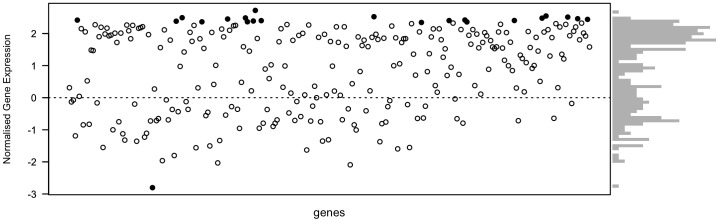
The effect of signal combinations differs from the sum of their effects in isolation. The expression profile of 264 QS regulated genes are plotted, comparing the sum of the individual effects of two signals (C4-HSL and 3-oxo-C12-HSL) with the effect of adding them in combination. The dotted line at 0 represents the summed null expectation and the points represent the difference between the null expectation and the effect of adding both signals. The points are coloured in black if they differ from the null expectation more than would be expected by chance (see Materials and Procedure). In 17 of these the effect of adding both signals exceeded the summed expectation and in 1 case the summed expectation exceeded the observed expression. Gene expression is normalised per gene and gene expression is given in units of standard deviation. The histogram on the right represents the distribution of differences between the null expectation (sum) and the effect of adding both signals.

We found that QS regulated the expression of 264 genes. This means that expression was significantly altered by at least one signal addition treatment (C4-HSL, 3-oxo-C12-HSL or Both). To test wether the sum of the individual effects (additivity) differ from the combined effect, for any of these genes, we compared the deviance from additivity to an empirically derived null distribution (see Methods & Materials for details). We found that the number of genes for which the null assumption was violated was higher than would be expected by chance given an alpha of 0.05 (Binomial test, p<0.01, probability  = 0.091, 95% CI: 0.058–0.134). After accounting for multiple testing, we identified 18 candidate genes that significantly deviated from the additive expectation (Figure 2, black dots). In 17 the summed individual effects gave a lower expected expression than observed but in one case the summed effects exceeded that of the combined effect (Figure 2). This indicates that the signal combination can be processed in at least two different ways (see also [31]).

Put simply, the effect of perceiving both signals at once differs significantly from that which would be expected given sum of the individuals effects of each signal. This is the same general pattern of results as the studies of combinatorial communication in putty-nosed monkeys: the effect of the combined signal differs from the composite effect of the two individual signals.

## Discussion

These results show that when presented simultaneously with two known signals, bacteria respond in a way that is different to the sum of the responses to the two individual signals. How should this finding be interpreted? The most cautious interpretation would be that it is merely an incidental side-effect of mechanisms that have other functions. To illustrate, if you hear a horn and a bell at the same time, your likely reaction would be something different to the sum of your separate reactions to the horn and to the bell - but this would not mean that the horn and the bell together is a combinatorial signal. The reason it would not is that: (i) horns and bells are not regularly heard together; and, in particular (ii) there is no reason to suppose that the co-production of horns and bells is anything more than incidental. However in this case, (i) is not true (as mentioned above, 3-oxo-C12-HSL and C4-HSL are both produced together in vitro and found together in vivo), and (ii) is unlikely to be true: a number of observations collectively suggest that the co-production of 3-oxo-C12-HSL and C4-HSL in nature is more than incidental. In particular: QS molecules are always produced as long as there are substrates to do so and as long as the regulatory inputs are functional (in other words, 3-oxo-C12-HSL and C4-HSL are standardly produced together); the rate of production of 3-oxo-C12-HSL and C4-HSL is a function of the state of the local environment; and the systems for production and reception (lasIR and rhlIR) are simultaneously active over a broad range of environmental conditions i.e. production and reception coincide [21]. In sum, co-production occurs often, is environmentally determined, and takes place in the presence of mechanisms designed for reception (ibid.). Given this, a cautious (‘horn-and-bell’) interpretation of our results, whilst plausible, seems improbable. It is more likely that the combination of the two signals is itself a (composite) signal. (Note that this is the same type of reasoning as that used in the study of combinatorial communication in non-human primates. There too, the horn-and-bell objection is refuted by observations about the circumstances in which the purported composite signal is observed in nature, which make clear that production is not incidental [7].)

The immediate implication of our findings is that relative levels of cognitive sophistication cannot explain the distribution of combinatorial communication in the natural world. This should perhaps not be a surprise: there is no particular reason to believe that signal combinations should in and of themselves be cognitively demanding. It has simply been assumed that they are cognitively demanding, but no argument has been given for this claim. More generally, our results raise the possibility that combinatorial communication is more common than has previously been assumed.

Further implications are harder to discern, and in many respects that is the point. Communication systems cannot be compared directly in the way that biological traits normally can, because, being systems, they are not themselves biological traits subject to natural selection [32]. They are instead the product of two interacting traits, namely mechanisms for production and mechanisms for reception [ibid.]. This fact has two serious implications for comparative research. First, it means that the communication systems cannot, by definition, be evolutionary analogues or homologues of one another; only mechanisms for production and reception can be. Second, the interactivity of these mechanisms places serious constraints on whether and how combinatorial systems can evolve, to the extent that combinatorial communication is expected to be rare in general, regardless of any supposed cognitive challenges it might present [33], [34]. Consequently, it is not clear how comparisons between communication systems should in general be interpreted. This point applies not only to signal combinations, but also to other surface qualities of communication systems, for instance referentiality. This is not at all to suggest that cross-species comparisons are irrelevant; only that when making such comparisons we must be cognisant of the fact that we are not comparing biological traits directly, and that this fact must be borne in mind in our conclusions.

We also suggest in particular that research that compares non-human primate communication with language should focus less on the surface form of those communication systems, but rather on whether the communicative behaviour of different species is based upon the same underlying cognitive mechanisms as linguistic communication, or different ones [e.g. 35]. In particular, linguistic communication is a special and important form of ostensive communication i.e. of the expression and recognition of intentions [34,36; other forms of ostensive communication include pointing, shrugging, eye gaze, and so on]. As such, in order to understand the evolutionary origins of human communication and language, the most insightful comparisons will be with the cognitive mechanisms that make ostensive and hence linguistic communication possible in the first place, rather than with the surface form of the different communication systems [34], [37]. Some research in this direction has taken place [35], [38], but more can and should be pursued.

## Methods & Materials

We analysed data from a parallel study on Quorum Sensing in P. aeruginosa [31]. A double QS synthase mutant of Pseudomonas aeruginosa PAO1 lasI/rhlI was grown at 37°C in 25 ml LB broth and 250 ml flasks with shaking at 200 r.p.m. Where required, LB broth was supplemented with 15 µM QS signal(s) in the following four treatments: (a) no addition; (b) 3-oxo-C12-HSL; (c) C4-HSL; and (d) both 3-oxo-C12-HSL and C4-HSL. Two replicate cultures were used for each treatment.

RNA was extracted from each culture after 8 h incubation (late exponential/early stationary phase of growth). Cells were treated with RNAprotect Bacteria Reagent (Qiagen), and total RNA extraction was performed with the RNeasy Midi Kit (Qiagen) as per the manufacturer's instructions.

For the expression profiling experiments, the microarrays were designed to contain multiple oligonucleotide probes for all the PAO1 genes including the small RNA genes and were purchased from Oxford Gene Technology (Oxford, UK). For each array, 10 ug of RNA was reverse transcribed and directly labelled with Cy5-dCTP and 2 ug of genomic DNA was directly labelled with Cy3-dCTP. Samples were hybridized onto the arrays for 16 h. Scanning of the arrays was performed using the Axon 4000B GenePix Scanner, the data extraction software used was GenePix Pro 6, both from Molecular Devices (Sunnyvale, USA).

For each treatment, microarray experiments were performed in duplicate and data capture was performed using GeneSpring GX10 (Agilent Technologies, Santa Clara, USA). Further data analysis was performed using the linear models for microarray data (‘limma’) package on the open source statistical platform ‘R’ (v2.14.2). Following Quantile normalization, differential expression was identified using Bayesian adjusted t-statistics with false discovery rate correction for multiple testing. In all comparisons (every signal addition treatment vs. the control of no signal), the criterion for differential expression was an false discovery rate corrected p value of less than 0.05. Using this method we identified 264 genes that were regulated by QS.

To test whether any significantly regulated genes were ‘combinatorial’ we evaluated the null hypothesis that the sum of the individual effects would be equal to the observed effect of adding both signals. We first normalised expression per gene and then for each gene, we compared the observed deviance (squared difference between adding both signals and the summed expectation) to the null deviance distribution derived by permutation. We then asked whether the observed value was in the within the 95% limit of the null distribution. We did this with 10,000 permutations and for each gene counted in what proportion of permutations did the observed value fall in 95% limit (equal the p value). We then tested whether the number of genes with a p value of less than 0.05 was greater than expected by chance using a binomial test with alpha  = 0.05. Finally p values were corrected for multiple testing via the ‘holm’ method to reduce the incidence of false positives [39]. Using this approach we identified 18 genes that deviated significantly from the sum expectation (Figure 2).

The data reported here have been uploaded to the Gene Expression Omnibus (GEO, www.ncbi.nlm.nih.gov/geo/) with the accession number GSE55110.

## References

[1] von Frisch K (1967) The dance language and orientation of bees. Cambridge, MA: Belknap Press.

[2] Hockett CF (1960) The origin of speech. Sci Am 203: 88–96. 14402211

[3] Fitch WT (2010) The evolution of language. Cambridge: Cambridge University Press.

[4] Hurford JR (2011) The origins of grammar. Oxford: Oxford University Press.

[5] Chomsky N (1965) Aspects of the theory of syntax. Cambridge, MA: MIT Press.

[6] Maynard Smith J, Szathmáry E (1995) The major transitions in evolution. Oxford: Oxford University Press.

[7] Arnold K, Zuberbühler K (2006) Language evolution: Semantic combinations in primate calls. Nature 441: 303. 16710411 10.1038/441303a

[8] Arnold K, Zuberbühler K (2008) Meaningful call combinations in a non-human primate. Curr Bio 18: R202–R203. 18334192 10.1016/j.cub.2008.01.040

[9] Zuberbühler K (2002) A syntactic rule in forest monkey communication. Animal Behaviour 63: 293–299.

[10] Ouattara K, Lemasson A, Zuberbühler K (2009) Campbell's monkeys concatenate vocalizations into context-specific call sequences. Proc Natl Acad Sci USA 106: 22026–22031. 20007377 10.1073/pnas.0908118106 PMC2799830

[11] Schuster M, Greenberg EP (2006) A network of networks: Quorum-sensing gene rgulation in Pseudomonas aeruginosa. Int J Med Microbiol 296: 73–81. 16476569 10.1016/j.ijmm.2006.01.036

[12] Williams P, Winzer K, Chan WC, Cámara M (2007) Look who's talking: Communication and quorum sensing in the bacterial world. Phil Trans R Soc B 362: 1119–1134. 17360280 10.1098/rstb.2007.2039 PMC2435577

[13] Ng WL, Bassler BL (2009) Bacterial quorum-sensing network architectures. Annu Rev Genet 43: 197–222. 19686078 10.1146/annurev-genet-102108-134304 PMC4313539

[14] Darch S, West S, Winzer K (2012) Density-dependent fitness benefits in quorum-sensing bacterial populations. Proc Natl Acad Sci USA 109: 8259–8263. 22566647 10.1073/pnas.1118131109 PMC3361460

[15] Maynard Smith J, Harper DGC (2003) Animal Signals. Oxford: Oxford University Press.

[16] Scott-Phillips TC (2008) Defining biological communication. J Evo Biol 21: 387–395. 10.1111/j.1420-9101.2007.01497.x 18205776

[17] Diggle SP, Gardner A, West SA, Griffin AS (2007) Evolutionary theory of bacterial quorum sensing: when is a signal not a signal? Phil Trans R Soc Lond B 362: 1241–1249. 17360270 10.1098/rstb.2007.2049 PMC2435587

[18] Pearson JP, Pesci EC, Iglewski BH (1997) Roles of Pseudomonas aeruginosa las and rhl quorum-sensing systems in control of elastase and rhamnolipid biosynthesis genes. J Bacteriol 179: 5756–5767. 9294432 10.1128/jb.179.18.5756-5767.1997 PMC179464

[19] Pesci EC, Pearson JP, Seed PC, Iglewski BH (1997) Regulation of las and rhl quorum sensing in Pseudomonas aeruginosa. J Bacteriol 179: 3127–3132. 9150205 10.1128/jb.179.10.3127-3132.1997 PMC179088

[20] Singh PK, Schaefer AL, Parsek MR, Moninger TO, Welsh MJ, Greenberg EP (2000) Quorum-sensing signals indicate that cystic fibrosis lungs are infected with bacterial biofilms. Nature 407: 762–764. 11048725 10.1038/35037627

[21] Duan K, Surette MG (2007) Environmental Regulation of Pseudomonas aeruginosa PAO1 Las and Rhl Quorum-Sensing Systems. J Bacteriol 189: 4827–4836. 17449617 10.1128/JB.00043-07 PMC1913434

[22] Whiteley M, Lee KM, Greenberg EP (1999) Identification of genes controlled by quorum sensing in Pseudomonas aeruginosa. Proc Natl Acad Sci USA 96: 13904–13909. 10570171 10.1073/pnas.96.24.13904 PMC24163

[23] Whiteley M, Greenberg EP (2001) Promoter specificity elements in Pseudomonas aeruginosa quorum-sensing-controlled genes. J Bacteriol 183: 5529–5534. 11544214 10.1128/JB.183.19.5529-5534.2001 PMC95443

[24] Wagner VE, Bushnell D, Passador L, Brooks AI, Iglewski BH (2003) Microarray analysis of Pseudomonas aeruginosa quorum-sensing regulons: Effects of growth phase and environment. J Bacteriol 185: 2080–2095. 12644477 10.1128/JB.185.7.2080-2095.2003 PMC151498

[25] Schuster M, Lostroh CP, Ogi T, Greenberg EP (2003) Identification, timing, and signal specificity of Pseudomonas aeruginosa quorum-controlled genes: A transcriptome analysis. J Bacteriol 185: 2066–2079. 12644476 10.1128/JB.185.7.2066-2079.2003 PMC151497

[26] Schuster M, Greenberg EP (2007) Early activation of quorum sensing in Pseudomonas aeruginosa reveals the architecture of a complex regulon. BMC Genomics 8: 287. 17714587 10.1186/1471-2164-8-287 PMC2018727

[27] de Kievit TR, Kakai Y, Register JK, Pesci EC, Iglewski BH (2002) Role of the Pseudomonas aeruginosa las and rhl quorum-sensing systems in rhlI regulation. FEMS Microbiol Lett 212: 101–106. 12076794 10.1016/s0378-1097(02)00735-8

[28] Pesci EC, Iglewski BH (1997) The chain of command in Pseudomonas quorum sensing. Trends Microbiol 5: 132–4. 9141185 10.1016/S0966-842X(97)01008-1

[29] Diggle SP, Winzer K, Chhabra SR, Worrall KE, Cámara M, Williams P (2003) The Pseudomonas aeruginosa quinolone signal molecule overcomes the cell density-dependency of the quorum sensing hierarchy, regulates rhl-dependent genes at the onset of stationary phase and can be produced in the absence of LasR. Mol Microbiol 50: 29–43. 14507361 10.1046/j.1365-2958.2003.03672.x

[30] Dekimpe V, Déziel E (2009) Revisiting the quorum-sensing hierarchy in Pseudomonas aeruginosa: the transcriptional regulator RhlR regulates LasR-specific factors. Microbiol 155: 712–723. 10.1099/mic.0.022764-0 19246742

[31] Cornforth DM, Popat R, McNally L, Gurney J, Scott-PhillipsTC, et al. (in press). Combinatorial quorum-sensing allows bacteria to resolve their social and physical environment. Proc Nat Acad Sci. 10.1073/pnas.1319175111 PMC3964068 24594597

[32] Scott-Phillips TC, Blythe RA, Gardner A, West SA (2012) How do communication systems emerge? Proc Roy Soc B 279: 1943–1949. 10.1098/rspb.2011.2181 PMC3311886 22217724

[33] Scott-Phillips TC, Blythe RA (2013) Why is combinatorial communication rare in the natural world, and why is language an exception to this trend? J Roy Soc Int 10 (88). 10.1098/rsif.2013.0520 PMC3785817 24047871

[34] Scott-Phillips TC (in press) Speaking Our Minds. London: Palgrave MacMillan.

[35] Tomasello M (2008) Origins of Human Communication. Cambridge, MA: MIT Press.

[36] Sperber D, Origgi G (2010) A pragmatic perspective on the evolution of language. In: Larson RK, Déprez V, Yamakido H, editors. The Evolution of Human Language: Biolinguistic Perspectives. Cambridge: Cambridge University Press. pp. 124–131.

[37] Scott-Phillips TC (in revision) Non-human primate communication, pragmatics, and the origins of language. Curr Anth.

[38] Moore R, Mueller B, Kaminski J, Tomasello M, (in revision) Two-year-olds but not domestic dogs (Canis familiaris) understand communicative intentions without language, gestures, or gaze. Dev Sci. 10.1111/desc.12206 25041186

[39] Holm S (1979) A simple sequentially rejective multiple test procedure. Scan J Stat 6: 65–70.

